# Enhanced Cohort Methods for HIV Research and Epidemiology (ENCORE): Protocol for a Nationwide Hybrid Cohort for Transgender Women in the United States

**DOI:** 10.2196/59846

**Published:** 2024-08-27

**Authors:** Andrea L Wirtz, Tonia Poteat, Annick Borquez, Sabriya Linton, Megan Stevenson, James Case, Carter Brown, Arianna Lint, Marissa Miller, Asa Radix, Keri N Althoff, Jason S Schneider, J Sonya Haw, Andrew J Wawrzyniak, Allan Rodriguez, Erin Cooney, Elizabeth Humes, Ceza Pontes, Shannon Seopaul, Camille White, Chris Beyrer, Sari L Reisner, ENCORE Study Group

**Affiliations:** 1 Department of Epidemiology, Johns Hopkins Bloomberg School of Public Health Baltimore, MD United States; 2 Department of International Health, Bloomberg School of Public Health, Johns Hopkins University Baltimore, MD United States; 3 Department of Public Health Sciences, Dalla Lana School of Public Health, University of Toronto Toronto, ON Canada; 4 Division of Healthcare in Adult Populations, Duke University School of Nursing Durham, NC United States; 5 Division of Infectious Diseases and Global Public Health, University of California San Diego La Jolla, CA United States; 6 Department of Mental Health, Bloomberg School of Public Health, Johns Hopkins University Baltimore, MD United States; 7 Johns Hopkins School of Nursing Baltimore, MD United States; 8 National Black Transgender Advocacy Coalition Carrolton, TX United States; 9 Arianna's Center Fort Lauderdale, FL United States; 10 Trans Solutions Research and Resource Center Indianapolis, IN United States; 11 Callen-Lorde Community Health Center New York, NY United States; 12 Division of General Internal Medicine, Department of Medicine, Emory University Atlanta, GA United States; 13 Division of Endocrinology, Metabolism and Lipids, Department of Medicine, Emory University Atlanta, GA United States; 14 Department of Psychiatry and Behavioral Sciences, University of Miami Miller School of Medicine Miami, FL United States; 15 Division of Infectious Diseases, Department of Medicine, University of Miami Miller School of Medicine Miami, FL United States; 16 Department of International Health, Johns Hopkins Bloomberg School of Public Health Baltimore, MD United States; 17 Duke Global Health Institute, Duke University Durham, NC United States; 18 Department of Epidemiology, University of Michigan School of Public Health Ann Arbor, MD United States; 19 See Acknowledgments

**Keywords:** transgender women, cohort, United States, HIV, digital methods

## Abstract

**Background:**

In the United States, transgender women are disproportionately impacted by HIV and prioritized in the national strategy to end the epidemic. Individual, interpersonal, and structural vulnerabilities underlie HIV acquisition among transgender women and fuel syndemic conditions, yet no nationwide cohort monitors their HIV and other health outcomes.

**Objective:**

Our objective is to develop a nationwide cohort to estimate HIV incidence, identify risk factors, and investigate syndemic conditions co-occurring with HIV vulnerability or acquisition among US transgender women. The study is informed by the Syndemics Framework and the Social Ecological Model, positing that stigma-related conditions are synergistically driven by shared multilevel vulnerabilities.

**Methods:**

To address logistical and cost challenges while minimizing technology barriers and research distrust, we aim to establish a novel, hybrid community hub–supported digital cohort (N=3000). The digital cohort is the backbone of the study and is enhanced by hubs strategically located across the United States for increased engagement and in-person support. Study participants are English or Spanish speakers, are aged ≥18 years, identify as transgender women or along the transfeminine spectrum, reside in 1 of the 50 states or Puerto Rico, and do not have HIV (laboratory confirmed). Participants are followed for 24 months, with semiannual assessments. These include a questionnaire and laboratory-based HIV testing using self-collected specimens. Using residential zip codes, person-level data will be merged with contextual geolocated data, including population health measures and economic, housing, and other social and structural factors. Analyses will (1) evaluate the contribution of hub support to the digital cohort using descriptive statistics; (2) estimate and characterize syndemic patterns among transgender women using latent class analysis; (3) examine the role of contextual factors in driving syndemics and HIV prevention over time using multilevel regression models; (4) estimate HIV incidence in transgender women and examine the effect of syndemics and contextual factors on HIV incidence using Poisson regression models; and (5) develop dynamic, compartmental models of multilevel combination HIV prevention interventions among transgender women to simulate their impact on HIV incidence through 2030.

**Results:**

Enrollment launched on March 15, 2023, with data collection phases occurring in spring and fall. As of February 24, 2024, a total of 3084 individuals were screened, and 996 (32.3%) met the inclusion criteria and enrolled into the cohort: 2.3% (23/996) enrolled at a hub, and 53.6% (534/996) enrolled through a community hub–supported strategy. Recruitment through purely digital methods contributed 61.5% (1895/3084) of those screened and 42.7% (425/996) of those enrolled in the cohort.

**Conclusions:**

Study findings will inform the development of evidence-based interventions to reduce HIV acquisition and syndemic conditions among US transgender women and advance efforts to end the US HIV epidemic. Methodological findings will also have critical implications for the design of future innovative approaches to HIV research.

**International Registered Report Identifier (IRRID):**

DERR1-10.2196/59846

## Introduction

### Background

Transgender women, broadly inclusive of those who identify as transgender women or along the transfeminine spectrum, experience a disproportionately high prevalence of HIV and are prioritized in the Ending the HIV Epidemic (EHE) and National HIV Strategy in the United States [1-3]. Multiple biological, behavioral, and interpersonal vulnerabilities for HIV infection among transgender women are driven and reinforced by structural barriers that limit access to HIV prevention, testing, care, and health services [4]. Transgender women in the United States experience an estimated 14% laboratory-confirmed HIV prevalence [2], although estimates as high as 42% have been reported in recent HIV surveillance, with significant disparities across minoritized racial and ethnic groups [5]. Recent estimates from a cohort of transgender women in Eastern and Southern United States reported an HIV incidence rate of 5.5 per 1000 person-years; there were notable disparities by race and ethnicity, with the incidence rate being as high as 19.3 per 1000 person-years among Black participants [6].

Understanding HIV acquisition among transgender women must be considered in the context of syndemic conditions, that is, “the concentration and deleterious interaction of two or more diseases or other health conditions in a population, especially as a consequence of social inequity and the unjust exercise of power” [7], including the high prevalence of mental health conditions, substance use disorders, and structural and interpersonal violence as a consequence of stigma and discrimination [8-28]. Findings from the National HIV Behavioral Surveillance indicated that among transgender women, more syndemic conditions were associated with increased condomless anal intercourse across racial and ethnic groups, and the prevalence of different syndemic conditions varied by racial and ethnic groups, with excess burden among Black and Latina transgender women [29]. Cross-sectional research has also demonstrated the co-occurring and deleterious interactions of syndemic conditions with HIV vulnerability and with adherence to antiretrovirals for HIV prevention and care [30,31]. Alongside risks, it is vital to identify health-promoting factors and resiliencies that may be leveraged for interventions, including gender affirmation, community involvement, and social support [32-34]. Collectively, this work emphasizes the importance of developing HIV prevention strategies for transgender women that address structural and psychosocial syndemic conditions, support resiliencies, and are tailored for specific demographic groups.

National cohorts are also critical to monitoring epidemic trends and how major events (eg, epidemics, policy changes, and new biomedical interventions) impact HIV and other health conditions, particularly for transgender women who are often misclassified with other populations. While transgender women have contributed extensively to domestic HIV research, most studies in the United States have been cross-sectional or subsumed transgender women within broader risk groups [35-37], limiting scientific inference. Only two cohorts have been implemented among transgender women exclusively in the United States: (1) a facility-based international cohort study of transgender women conducted from 2017 to 2019 that included a site in San Francisco [38] and (2) a multisite, multimodal cohort study conducted from 2018 to 2022 to assess HIV incidence exclusively among transgender women in Eastern and Southern United States (the LITE cohort) [39,40]. Sustained cohorts focused on transgender women are needed to monitor epidemic trends unique to transgender women and to assess whether and how national health policies and HIV prevention efforts, including new pre-exposure prophylaxis (PrEP) modalities, impact HIV acquisition in transgender women.

Irrespective of the study population, contemporary HIV cohorts are either facility based or exclusively digital in implementation. Few to none of the cohorts evaluate both models or test hybrid approaches, and most digital cohorts neglect to measure technology access and literacy, missing an opportunity to assess potential biases or test approaches that build on the efficiencies of both models. Results from our prior cohort showed that participants in the site-based mode were more likely to experience individual and structural vulnerabilities (eg, unemployment, sex work, and drug use), whereas participants in the digital mode reported psychosocial and other health vulnerabilities (eg, social isolation, low access to gender-affirming care, and hazardous alcohol use) [6]. Through community engagement, the site-based technology-enhanced model allowed our team to overcome research distrust and permitted follow-up in local communities, revealing incarcerations and deaths that may have been missed in exclusively digital (web-based) cohorts [6]. Mitigating selection bias and the attrition of vulnerable populations is also important for an epidemic underscored by social and health disparities. We aim to explore whether hybrid approaches that leverage the strengths of site-based cohort models can facilitate the engagement and retention of diverse participants in digital HIV cohorts.

### Objectives

Our overarching objectives are two-fold: (1) to develop and assess a hybrid community hub–supported digital cohort model, drawing on the unique strengths of site-based and digital cohort methods, for engaging populations facing health, social, and structural contextual disparities, and (2) to evaluate the impact of structural and psychosocial syndemic experiences on HIV incidence among a nationwide cohort of transgender women to inform multilevel HIV prevention strategies.

Our study aims are to (1) determine the efficiency and acceptability of a novel, hub-supported digital cohort of racially and ethnically diverse transgender women aged ≥18 years who are HIV negative; (2) estimate the prevalence and characterize patterns of syndemic experiences among transgender women; (2.1) examine the role of contextual factors (virtual and physical) in driving syndemic experiences over time among transgender women using latent class trajectory analysis; (3) estimate HIV incidence in transgender women, followed every 6 months for at least 24 months to identify tailored approaches for multilevel combination HIV prevention interventions; (3.1) examine the effect of syndemic experiences and contextual factors on HIV incidence among transgender women in the United States; (3.2) characterize the PrEP continuum among transgender women and associations with HIV incidence over time, including the uptake of newly emerging formulations; longitudinal patterns of HIV risk and adherence; and the role of syndemic classes and contextual factors in PrEP uptake, adherence, and retention; and (4) develop dynamic models of multilevel combination HIV prevention interventions and scale-up among transgender women to simulate the impact of evaluated interventions on HIV incidence through 2030, corresponding to the timeline for ending the HIV epidemic outlined in the National HIV strategy.

## Methods

### Overview

This study is led by principal investigators (PIs) from Johns Hopkins University (PI ALW) and University of Michigan (PI SLR). The study is supported by leading transgender-led, community-based organizations, namely Arianna’s Center, Trans Solutions, and the Black Trans Advocacy Coalition, and leading research institutions across the United States, including Johns Hopkins University, Baltimore, Maryland; University of Michigan, Ann Arbor, Michigan; Callen-Lorde, New York City, New York; Emory University and Grady Memorial Hospital, Atlanta, Georgia; University of Miami, Miami, Florida; University of California San Diego, San Diego, California; and Duke University, Durham, North Carolina. Community coinvestigators are essential to the study as they (1) provide input across study design and implementation; (2) serve as bridges to community partners operating in counties outside of hub sites; and (3) support study recruitment, retention, and dissemination across community networks. The coinvestigator research institutions have expertise in HIV epidemiology among key populations, transgender health and clinical care, the management and analysis of longitudinal cohorts, HIV modeling, and sociobehavioral sciences. All study sites are affiliated with or are clinical sites serving transgender women, and all have long established relationships with local organizations and the trans community. A community advisory board (CAB) supports the study team by (1) providing guidance and feedback on research activities and materials; (2) discussing concerns and protection of the community; (3) supporting dissemination efforts within the wider transgender women community; and (4) ensuring the study is conducted in an appropriate and acceptable manner, as well as ensuring the objectives and reporting are beneficial to transgender women.

### Conceptual Framework

This proposal is informed by 2 conceptual frameworks, the Syndemics Framework and the Social Ecological Model. Syndemics have been defined as “the concentration and deleterious interaction of two or more diseases or other health conditions in a population, especially as a consequence of social inequity and the unjust exercise of power” [7]. Our conceptualization of social inequity uses an intersectional lens, recognizing that social categories (eg, race, ethnicity, and gender) intersect to affect individual experiences via systems of power and oppression (ie, racism, sexism, and transphobia) [41,42]. For example, among transgender women, pervasive stigma and discriminatory laws compel engagement in sex work for economic survival and gender affirmation while also creating barriers to the provision and uptake of HIV services [32,43,44]. We seek to understand how these and other conditions of social inequity contribute to HIV vulnerability and drive co-occurring, synergistic health conditions. We also seek to identify and understand population strengths and resilience factors (eg, social support, community involvement, and gender affirmation) that may mitigate the social inequities that drive HIV syndemics and inform interventions to improve HIV prevention outcomes.

We apply the Syndemics Framework with the Social Ecological Model in recognition that multiple and intersecting levels of risk shape the interaction and distribution of HIV risk among transgender women [45,46]. The Social Ecological Model considers the complex interplay among individual, interpersonal, and structural factors that impact health [47]. We focus on how syndemic conditions at the individual, interpersonal, and structural levels co-occur with the HIV epidemic and may potentiate HIV acquisition risk [48]. The impact of age, race, and ethnicity at the individual level reflects systems of oppression. At the individual level, substance use and mental health disorders reduce the uptake of and adherence to HIV prevention strategies and increase risk for violence [49]. Interpersonal risks include intimate partner violence (IPV), law enforcement interaction, and sex work [50,51]. Employment and health care access, the availability of safe and affordable housing, and exposure to safe environments likely impact health at the structural level.

### Recruitment

Our recruitment strategies are built on prior experiences [39,40] and strategies successful in other studies [51-56]. Strategies include community-based recruitment methods (tabling at events, distributing flyers at community organizations, word of mouth, peer referral, etc) as well as web-based methods (app-based advertisements, Google Ads [Google LLC], community listserves, closed social media groups, etc). Given disparities in HIV by race, ethnicity, and geographic location, we ensured diversity by providing recruitment materials in English and Spanish, using diverse images, and implementing geotargeted advertising to southern United States and EHE priority areas.

### Study Population

The cohort is composed of English- or Spanish-speaking adults at risk for HIV, aged ≥18 years, assigned male sex at birth who identify as women or along the transfeminine spectrum, and living in the United States or Puerto Rico. Gender identity is verified during enrollment using the 2-step method measuring sex designated at birth (step 1) and current gender identity (step 2) [57,58]. Enrollment in the cohort is restricted to participants with laboratory-confirmed negative HIV test results at enrollment. We do not use stricter inclusion criteria related to sexual risk (eg, PrEP indication), given our prior findings that sexual behaviors are dynamic [59]. An exclusive focus on PrEP-indicated transgender women at baseline misses opportunities to investigate factors that predict changes in sexual behaviors that affect HIV vulnerability. Given documented disparities in the HIV epidemic, our engagement strategy included (1) recruitment efforts using intensive geotargeting to the southern United States; (2) hubs located in EHE priority counties [1]; and (3) a goal to enroll a study population in which at least 50% of participants identify as Black, Indigenous, or Latina.

Cohort participants complete self- or interviewer-administered registration activities, including a preliminary screening to assess eligibility based on age, gender, and self-reported HIV status. Preliminarily eligible participants complete a verification and are then prompted to complete the consent process, which is followed by registration and access to the baseline survey. Completing the initial survey triggers a laboratory order to ship the home specimen collection kit to participants, determined by the participants’ specimen collection method preference. Cohort enrollment is defined based on eligible screen, validation, consent, survey completion, and negative laboratory-based HIV test result.

Remote digital surveys are increasingly challenged by fraudulent entries by scammers and bots and duplicate entries [60]. We have built on best practices described by other investigators [35,61-63] and our own experiences to develop a comprehensive method to mitigate the risk of duplicate or fraudulent enrollments*.* These efforts include 2-step verification procedures, the exclusion of voice over IP (VOIP) phone numbers, real-time checks on duplicate information (phone, email, and IP address), restriction to US and Puerto Rico geographic IP addresses, and blocking banned IPs associated with spam and virus spread. Staff members review baseline survey data (eg, time to survey completion, missingness, and attention filter responses) and conduct phone interviews when fraudulence is suspected to verify that all participants meet preliminary eligibility and are legitimate.

### Hub-Supported Digital Cohort Model

**Figure 1:**
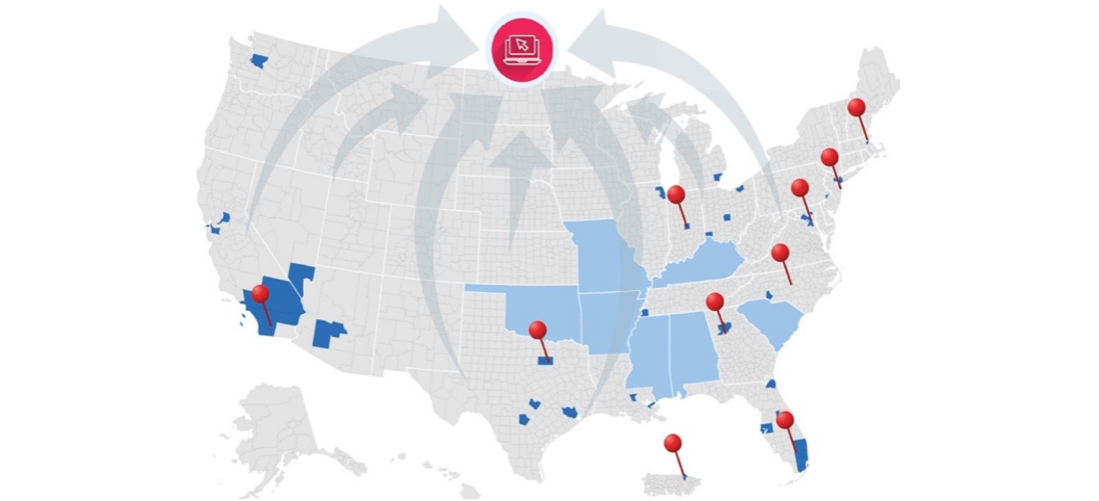
Conceptualization of hub-supported digital cohort with hubs (red) relative to Ending the HIV Epidemic (EHE) counties and states (dark and light blue).

A digital cohort platform serves as the backbone of this model wherein candidate participants can enroll and participate remotely from across all areas of the United States and Puerto Rico (Figure 1). Activities are fully technology-enabled, automated, and available in English and Spanish languages, including screening, consent, registration, HIV testing, peer referral, study reminders and notifications (except reactive or indeterminate HIV results), and incentive provision.

The nationwide, digital cohort is enhanced by community hubs strategically located across the United States. Hubs are not primarily responsible for implementing the study protocol or tracking participants but do support community-based recruitment and retention, engage the community via local collaborations, offer staff-administered screening and surveys for participants with low literacy or technology challenges, and support access to clinical services. Hub engagement can occur anywhere along the recruitment-enrollment-retention-referral continuum.

On the basis of prior work [39,40], we anticipate that approximately 65% to 70% of enrolled cohort participants will have fully digital participation, 20% to 25% will be recruited via hubs but will participate in a fully remote mode, and the remaining 5% to 10% of the cohort may require full or periodic in-person support. For those facing participation challenges outside of hub catchment areas, central staff members conduct screening, obtain consent, and administer surveys via phone.

### Study Visits

**Figure 2:**
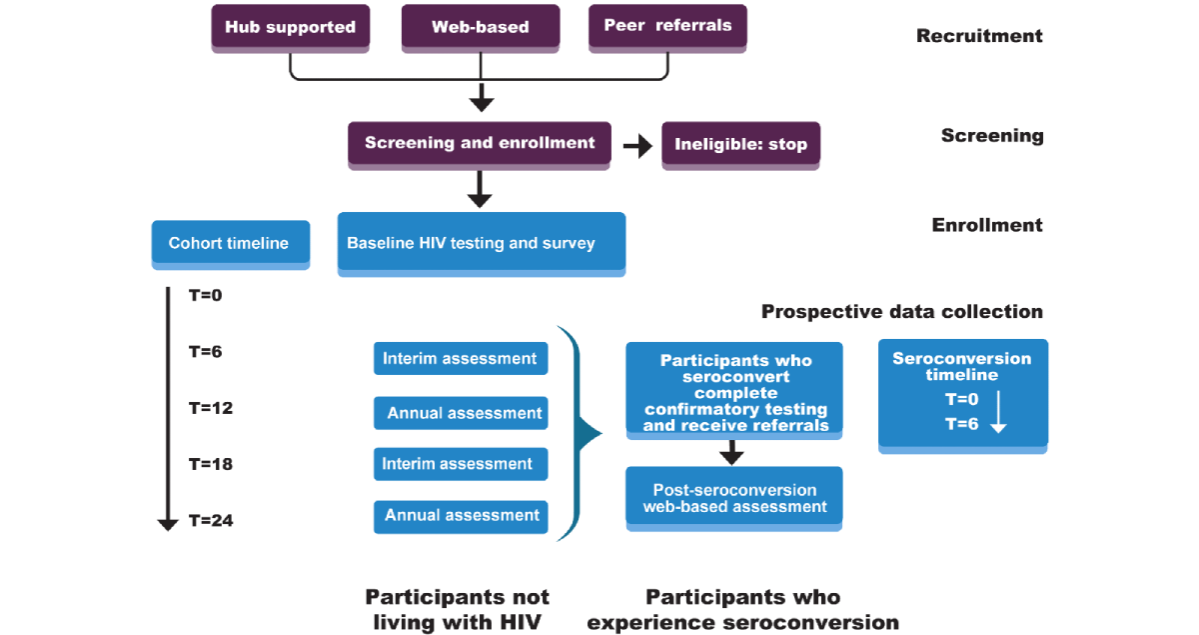
Flow diagram of the Enhanced Cohort Methods for HIV Research and Epidemiology (ENCORE) study.

Survey and laboratory data will be collected from cohort participants every 6 months for 24 months via web-based or hub-supported assessments using a web-based system (Figure 2). Participants who experience HIV seroconversion at any point during follow-up will be asked to complete a survey 6 months after confirmatory testing to assess their experiences with engagement in the HIV care continuum.

Study assessments include the completion of a survey questionnaire and HIV testing. Study staff members are available via telephone to support cohort participants with access to the web application. Automated emails and SMS text messages are sent to the participants’ device for reminders about upcoming and closing study assessments.

### Survey Measures

Survey measures are specific to the visit time point; each calendar year includes 1 fall and 1 spring visit. These calendar-based data collection periods allow for us to measure changes over time as well as conduct cross-sectional analyses for specific time periods. Core measures are repeated at each time point (income, sexual partnerships, mental health, PrEP use, etc), while others are asked either only at baseline (eg, static variables such as date of birth, country of origin, and adverse childhood experiences) or only once at a specific time point.

Survey measures include, but are not limited to, the following: individual-level measures include self-reported data on demographics, including the zip code of residence, age, race, ethnicity, gender identity, education, income, employment status, health insurance, housing, housing mobility, food insecurity, and history of detention or incarceration; gender affirmation surgery, care, and exogenous hormone use and interruptions, including the age of initiation, based on the US TransPop Survey [64]; sexual health history and the access or uptake of HIV services (including postexposure prophylaxis and PrEP) based on the Centers for Disease Control and Prevention’s National HIV Behavioral Surveillance [65]; primary care, mental or behavioral health, and barriers and facilitators to care [15,16,66]; substance use measured through the Alcohol Use Disorder Identification Test-Concise and the Drug Use Identification Test with additional measures for injecting drug use [67,68]; depression symptoms based on the Kessler Psychological Distress Scale and posttraumatic stress disorder (PTSD) symptoms based on the Primary Care PTSD scale [69-71]; and gender euphoria and transgender adaptation and integration assessing adjustment experiences specific to gender identity measured through the Gender Minority Stress and Resilience scale [72,73]. Childhood experiences are measured using the Adverse Childhood Experiences scale and Positive Childhood Experiences scale [74,75].

Interpersonal measures include sexual relationships and behaviors to assess self-reported detailed partner-by-partner sexual behavior information by partner type and HIV status, as well as substance use in the context of HIV acquisition risk, and has been adapted for trans populations to ensure gender-affirmative assessment of sexual risk (S Reisner, unpublished data, June 2017) [76]; and IPV and nonpartner violence victimization, including physical, sexual, and psychological violence and control, and abuse specific to one’s transgender experience (trans-specific IPV) [77].

Structural measures include social marginalization and stigma based on the Experiences of Discrimination scale adapted for trans adults in Massachusetts [78] and the US TransPop Survey (reasons for discrimination will be asked, eg, due to race, ethnicity, age, gender identity, or physical disability, in a “select-all-that-apply” format to examine identity and attributional intersectionalities) [64,79]. Social measures include community connectedness and pride using subscales from the Gender Minority Stress and Resilience scale, social support using an abbreviated version of the Medical Outcomes Study Social Support Survey, dignity using a scale developed by Khatib and Armenian [80], flourishing using a scale developed by Diener et al [81]; and experiences and perceptions of state-level stigmatizing and protective laws for transgender populations [73,82-84].

### HIV Testing

Biological specimen collection and laboratory-based HIV testing are conducted at every assessment following survey completion. We use self-collected dried blood spots (DBSs) as the primary method for HIV testing; however, based on formative research with LITE participants and feedback from the CAB, we offer several alternative HIV testing strategies to increase acceptability and decrease attrition associated with testing methodology.

The completion of each survey questionnaire automatically triggers the initiation of a laboratory requisition and shipment of a “home kit.” The home kit is a small box (ie, fits through all mail slots) that includes detailed, bilingual visual instructions for specimen collection and return mailing to the laboratory, as well as a resource page with information on HIV prevention services and other service options (eg, mental health support). Specimen self-collection materials include 4 lancets, alcohol wipes, adhesive bandage, and Whatman 5-spot DBS specimen card. Participants ship DBS specimens to the study laboratory, where they are tested by a College of American Pathologists–accredited and Clinical Laboratory Improvement Amendments–regulated laboratory using a fourth-generation HIV type 1 antigen/antibody enzyme-linked immunosorbent assay for the qualitative detection of HIV p24 antigen and antibodies to HIV type 1 (groups M and O) and HIV type 2 (sensitivity 100%; specificity 100%; Molecular Testing Labs, unpublished data, December 2020).

Participants who do not feel comfortable completing DBS specimen collection have the option to request oral HIV testing and are mailed an OraSure Oral Fluid Collection Device (OraSure Technologies) within 2 business days. The kits include an insert with study branding, a thank you message, addressed and stamped envelope for shipment to the laboratory, and instructions for oral fluid collection. HIV testing from the oral specimen is done via enzyme immune assay, with positive results being confirmed via western blot. Test performance is estimated to have a sensitivity of 99.6% and specificity of 99.9% [85].

Both DBS and oral fluid specimens are typically processed within 7 business days upon receipt by the laboratory. Notification of nonreactive HIV test results are automatically emailed to the participant. Staff members contact participants with a reactive result within 48 hours of receipt to provide posttest counseling and localized referrals for confirmatory testing and care. Participants near community hubs are linked to affirming services available at the hubs. Staff members call participants with an indeterminate result or rejected specimen to discuss the collection of another specimen or other testing options.

Participants who visit hubs to complete any study activities may also request to have a rapid HIV test completed on site according to local clinical protocols. Staff members enter rapid test results into the study data management system and provide any necessary referrals for confirmatory testing and services.

Participants who report recent testing outside of the study and within 30 days before or during the open study window may submit their HIV test results in lieu of HIV testing through the study. Participants are required to provide a copy of the laboratory report with the date of the laboratory test, laboratory test name, and identification of the participant. The primary purpose of this option is to reduce the research burden for participants who have had recent testing outside of the study.

### Contextual Data

By harmonizing cohort data with contextual, population-based data, our goal is to increase the knowledge base in this field and to parameterize mathematical models to estimate potential impacts of multilevel combination interventions on the HIV epidemic among transgender women. Using participants’ self-reported residential zip codes, person-level cohort data will be merged with contextual zip code–, county-, city-, and state-level data, including population health measures and economic, housing, and other social and structural factors (eg, protective laws for transgender populations and digital access; E Dworak, unpublished data, July 2020) [86-88]. Table 1 provides an example of the contextual, population-based data domains and sources that are considered for these analyses and the potential spatial areas where measures of a domain will be operationalized. Measures will be operationalized at a single level or multiple levels of geography depending on availability.

Contextual data will be updated annually or as available throughout the duration of follow-up to reflect the current HIV risk environment for transgender women in the United States. When appropriate, data will be interpolated for years when not available. We will also explore correlation among contextual data measures to determine whether data reduction techniques, such as principal component analysis, are necessary to summarize data measures.

**Table 1:** Population-based contextual data to be used in the Enhanced Cohort Methods for HIV Research and Epidemiology study.

Domain	Measures and definitions	Spatial area	Sources
Economic	Deprivation, polarization, inequality, and gentrification [20]	ZCTA^a^ or county	ACS^b^ [89]
Housing	Cost burden, inadequacy, unmet need for housing assistance, and racial and ethnic housing discrimination [3,90,91]	ZCTA or county	ACS [89,92], Picture of Subsidized Households [93], and Housing Mortgage Disclosure Act [90]
Social	Racial and ethnic polarization and segregation, drug-related law enforcement activity, social capital, and violent and hate crime rates	ZCTA or county	ACS [89] and FBI^c^ Uniform Crime Reports [91]
Health	HIV incidence, rates of opioid-involved overdose deaths, and density of behavioral health programs	ZCTA or county	AIDSVu [94], CDC WONDER^d^, National Directory of Drug and Alcohol Abuse Treatment Programs, and National Directory of Mental Health Treatment Facilities

^a^ZCTA: ZIP Code Tabulation Area.

^b^ACS: American Community Survey.

^c^FBI: Federal Bureau of Investigation.

^d^CDC WONDER: Centers for Disease Control and Prevention Wide-Ranging Online Data for Epidemiologic Research.

### Study Retention

The use of thorough tracking and retention procedures increases the likelihood that attrition will be random and not systematic. We use tracking and retention procedures proven effective in prior studies. Participants can update email addresses, phone numbers, and physical mailing addresses at any point to ensure contact information is as up to date as possible by logging into the web application. Contact information can also be updated by contacting study staff through email, phone, or text. Assessment reminders are sent to participants through telephone, email, or text once assessment windows open and periodically during the assessment window if the assessment has not been completed. Other strategies to improve retention include regular social events, both web-based and in person, such as study-sponsored yoga classes, cosmetics workshops, and an expungement clinic for marijuana or survival sex charges, as well as study team attendance at local community events.

Our research and community partners have excellent reputations within the community and are known for being nonstigmatizing and ensuring privacy and confidentiality, which assists with retention. We also have an extensive network of transgender-focused organizations and clinical care providers in each of our partnering metropolitan areas who serve as referral centers for health needs. We endeavor to hire study staff members who are transgender women or members of lesbian, gay, bisexual, transgender, and queer communities and who have nursing or public health experience to ensure cultural competence and optimize the acceptability of the study.

Retention is operationally defined as not missing >2 consecutive study assessments. After 1 missed assessment, staff members initiate the retention protocol to individually contact and track participants. Participants who express a desire to prematurely exit the study are asked to complete a participant withdrawal questionnaire (web-based, via phone, or in person) to assess basic characteristics at study exit (eg, HIV status and PrEP use), reason for withdrawal, and whether additional referrals are needed. The study team assesses whether the reason for withdrawal can be addressed and will offer resolution, within the limits of the protocol, to the participant. Aligned with our goals of a welcoming and supporting research environment and scientific rigor, cohort participants who are lost to follow-up or who have previously withdrawn and request to reenter the study will be screened and reassessed for eligibility and, if the timeline allows, administratively considered for re-enrollment.

### Sample Size Considerations

The measurement of HIV incidence in transgender women is the primary study outcome. With an enrollment goal of 3000 transgender women and an anticipated 10% loss to follow-up per year (as observed in our regional cohort), we estimate an accumulated 5130 person-years of follow-up when participants reach 24 months after enrollment. Given the HIV incidence rate of 5 per 1000 person-years in transgender women in our regional LITE cohort and the dramatic disparities involving a ≥3-fold increase in the rate in subgroups [6], this study has 80% power to detect a 2.6-fold difference in the HIV incidence rate by subgroups (eg, syndemic condition, race, and geography), assuming 2-sided test statistics and 5% type 1 error. In total, 75% of LITE participants reported at least 1 syndemic condition at baseline (eg, substance use, depression, or PTSD per the above criteria) [6]; thus, we expect that 2250 participants will report at least 1 condition, providing sufficient sample for subgroup analysis. Given the evidence of significant recruitment-enrollment cascades in digital cohorts [35,40], we anticipate 7000 individuals will be screened, 5000 will consent, and 3000 will have a negative baseline HIV result for full enrollment into the cohort.

### Analytic Plan

Analyses will explore efficiency (number of individuals enrolled and enrollment and retention rates); self-reported acceptability; and potential biases of those recruited, enrolled, and retained. We hypothesize that the hub-supported digital cohort will achieve the sample size and geographic representation goals within the first 24 months of the study. Participants recruited and retained by the hubs will be more racially and ethnically diverse and have lower technology access (neighborhood level and individual level) than those participating through exclusively digital means.

Aim-1 analyses will focus on evaluating the contribution of hub support to the digital cohort. Data visualization techniques will estimate time, cost, and response rates to explore the efficiency of the hub-supported digital cohort as a whole and compare time, cost, and response rates across modes (digital cohort only vs with hub support). Hub support will be identified by proxy links and self-reported exposure to hub activities. Quantitative descriptive analyses will include the comparison of measures of mode acceptability, participant characteristics (eg, demographics, risk behaviors, and HIV status), and contextual data (Table 1) to assess differences in the mode-specific samples that could impact epidemiologic inferences of HIV seroconversion rates. As a sensitivity analysis, we will repeat these analyses restricted to all participants residing in catchment areas around the hub locations. We will incorporate population-based contextual data pertaining to area-level broadband access to assess differences in enrollment and retention by these contextual features as well as personal technology use and access. Differences will be statistically compared across mode (eg, chi-square or Fisher exact test for differences in proportions); we will also use a minimally important “public health” difference. This difference, which is an adaptation of the minimal clinically important difference often used in clinical trials [95,96], is determined by investigators as the minimally meaningful difference that would have an impact on a public health measure, such as HIV prevention uptake. We will use a prespecified difference (eg, –5% and +5%) to incorporate the magnitude into our interpretations of differences, as we anticipate substantial heterogeneity by mode and process measures.

Aim-2 analyses will include latent class analyses of baseline cohort data to identify a set of discrete, mutually exclusive latent classes (syndemic “typologies”) of syndemic classes [97]. Latent class membership probabilities γ and item-response probabilities ρ will be estimated by maximum likelihood [97]. Each latent class will be characterized by a unique pattern of individual (eg, mental health and substance use), interpersonal (eg, violence victimization), and structural (eg, unstable housing and incarceration) vulnerabilities comprising syndemic conditions. Measurement invariance across groups will be tested by age, race, ethnicity, and geographic residence. Transgender women will be assigned to a class based on posterior probabilities. Multinomial regression models will estimate statistical predictors of class membership (eg, employment and health care access). HIV acquisition risk, operationalized as a composite variable of behavioral (sexual, injecting, and other behaviors) and risk environments associated with HIV in transgender women [98], will be regressed on latent classes to estimate the risk for one class relative to another (eg, testing associations between syndemic “typologies” and HIV risk) [99]. Model fit will be examined using appropriate indicators (eg, G^2^); model selection will be based on information criteria (eg, Akaike information criterion, Bayesian information criterion, consistent Akaike information criterion, and adjusted Bayesian information criterion) and interpretability [97,100,101].

We will examine syndemics over time and model trajectories of change in syndemic classes across follow-up with latent trajectory analyses and latent class growth analyses. We will model time-varying and -nonvarying predictors of changes in class membership, incorporating individual cohort and contextual data. Contextual data described in Table 1 will be incorporated into models to explore prospective impacts on syndemic experiences and differences by participant subgroups. Latent class analyses will use Mplus (Institute for Digital Research and Education).

Tracking and describing differences in attrition by individual, interpersonal, and structural risk factors will be essential to determining the appropriateness of assumptions in incidence and time-to-event analyses*.* Attrition will be tracked using data visualization techniques, stratifying important metrics over time (such as the number of individuals enrolled and proportion lost to follow-up) by site. Bimonthly, site-specific reports of attrition will be produced for review. We will alert staff to participants who are at risk to trigger a participant-focused retention protocol. Statistical analyses of attrition will include incidence rates and survival analyses to investigate rates of loss to follow-up and death overall among subgroups [102]. Risk ratios will be used to examine individual (eg, demographics and HIV risk behaviors), interpersonal (eg, social factors), and structural predictors of loss to follow-up and death [102].

HIV incidence rates will be estimated as the number of observed HIV seroconversions divided by the number of person-years accumulated; rate ratios and 95% CIs will be estimated using Poisson regression models [6]. Trends by 6-month time intervals will be monitored. HIV incidence rates measured in the entire cohort at 12 and 24 months will be estimated. In all trend analyses, a continuous variable for time in the Poisson regression model will test the null hypothesis that there is no difference (ie, no trend) in HIV incidence by 6- or 12-month interval of time since study entry. Assuming a sufficient number of events, a competing risks approach [103] will be used to account for the mortality. We will also visualize the Kaplan-Meier estimates of HIV cumulative incidence with a time-to-event approach that defines the time of origin as study entry and uses log-rank tests for differences, as well as Cox proportional hazards models to estimate hazard ratios and 95% CIs (standard for the competing risk of death) [104]. Analyses will be conducted overall and by subgroup.

Incorporating both cohort survey measures and contextual data, individual, interpersonal, and structural risk factors will be included in the Poisson regression models. To account for the nonindependence of observations within geographic areas, generalized estimating equations with a robust estimation of variances will be used to estimate the association of individual, interpersonal, and structural risk factors with HIV incidence [105]. We will separately model trajectories of HIV risk as a function of changing syndemic classes using latent trajectory analyses and latent class growth analyses.

Differences in PrEP indication and use among transgender women will be assessed at baseline. Perceptions and use of emerging formulations and modalities will be assessed using cross-sectional descriptive analyses of annual surveys at relevant time points. Descriptive statistics (chi-square tests and 2-tailed *t* tests) and log binomial models (or, when these models fail to converge, Poisson regression models with robust variance) will be used to estimate prevalence ratios and 95% CIs. Patterns of PrEP indication and prevention-relevant adherence over time will be estimated using group-based multitrajectory modeling, a data-driven approach for identifying latent longitudinal strata across distinct but related indicators (in separate models and using Nagin model selection criteria) [59,106,107]. We will then use the maximum posterior probability to assign participants into trajectory categories and use multinomial logistic regression models to identify correlates of each trajectory. Identified PrEP indication–adherence classes will be tested for associations with HIV incidence.

### Dynamic Compartmental Model

We will develop a dynamic compartmental model of HIV transmission and acquisition in transgender women and their sexual partners by incorporating information obtained through the Enhanced Cohort Methods for HIV Research and Epidemiology (ENCORE) cohort, the LITE cohort, and other prior work [108-111]. The model will be used to (1) better understand epidemic dynamics by quantifying the contribution of different individual, interpersonal, and structural determinants to HIV incidence in the context of other syndemic conditions (ie, substance use and mental health); (2) identify optimal PrEP regimen and delivery strategies; and (3) design HIV combination prevention packages to achieve EHE incidence reduction goals by 2030.

The model will disaggregate the population by key determinants of HIV risk and HIV care at the *individual level* (eg, age, race, ethnicity, and syndemic conditions), *interpersonal level (*eg, engagement in sex work and IPV), and *structural level* (health care access and employment). The population will also be disaggregated by HIV status or stage, HIV care status, and PrEP status. HIV transmission will be a function of biological and behavioral risk factors, including the number of partners, sex acts per partner, condom use and sexual positioning by partner type in each group, HIV prevalence and antiretroviral therapy (ART) coverage among partners, and PrEP coverage among susceptible individuals. The influence of individual, interpersonal, and structural determinants on HIV risk will be examined by applying relative risks to sexual behaviors associated with each determinant.

We will focus on 4 regional models: Northeast, South, Midwest, and West United States [112]. Nonetheless, it will be possible to apply the model in all US cities in the cohort by populating a database of city-specific model parameters that will be plugged into the region-specific calibration process. This will enable EHE cities to implement relevant analyses to guide local policy. Modeling will be conducted in collaboration with community coinvestigators and the CAB to ensure relevance and acceptability to the transgender women community.

For model parameterization, sociodemographic and behavioral parameters will be informed by the ENCORE cohort (all 4 regions) and the LITE cohort (northeastern and southern regions). Biological parameters defining HIV progression rates [113,114]; transmission probability for different HIV stages [113,114]; types of sex acts [115,116]; the effectiveness of condoms in preventing transmission during anal sex [117]; and the efficacy of ART and PrEP in preventing transmission [118] and acquisition [119,120], respectively, and in delaying ART HIV progression [121-123] will be obtained from systematic reviews and meta-analyses. Relative risks for the independent effect of each key determinant of HIV risk will be obtained through multivariable statistical models using the ENCORE data.

**Figure 3:**
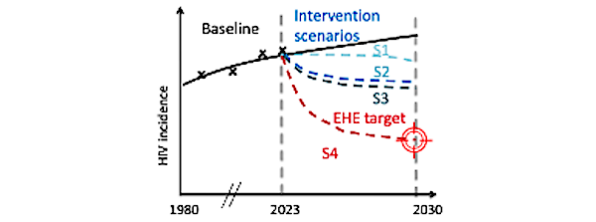
Predicted reduction in HIV incidence under different pre-exposure prophylaxis (PrEP) and combination prevention scenarios versus baseline. The black line corresponds to the baseline in the absence of PrEP and additional combination prevention interventions. EHE: Ending the HIV Epidemic.

In each of the 4 regions, we will carry out the following steps:

1. We will estimate the contribution of structural determinants (eg, unemployment and low health care access) to HIV incidence using a population attributable fraction (PAF) approach (by implementing counterfactual scenarios assuming no unemployment and adequate access to health care, respectively, over the past decade, 2013 to 2025). Using the same approach, we will estimate the PAF of IPV, substance use, and mental health disorders (in isolation and in combination) to characterize and quantify syndemic effects on the HIV epidemic. The proportion of new HIV infections averted in each scenario compared to the baseline scenario corresponds to the PAF of each determinant, allowing us to identify priorities for HIV prevention interventions [124,125].
2. We will identify PrEP delivery strategies by comparing a scenario with no PrEP with counterfactual scenarios of PrEP delivery. The status quo for PrEP delivery (Figure 3, line S1) will be based on current patterns of PrEP engagement and coverage. We will estimate the reduction in new HIV infections if PrEP patterns remain at current levels in each region from 2025 to 2030. A first counterfactual scenario will assume increased PrEP coverage by a relative 50% (Figure 3, line S2). A second counterfactual scenario will assume strategic PrEP provision based on characteristics identified in aim 4 (Figure 3, line S3). For example, those who engage in sex work might benefit the most from daily oral PrEP, while those experiencing substance use disorders or IPV might achieve better outcomes with long-acting formulations [126-128]. We will compare the number of new infections averted in each scenario to baseline (Figure 3, line S1) to assess the impact of increasing PrEP coverage and more strategic delivery.
3. We will design HIV combination prevention packages to achieve EHE incidence targets by identifying the most effective combination of interventions to achieve a 90% reduction in incidence among transgender women by 2030, compared to 2017 values in each region (Figure 3, line S4). In addition to PrEP scale-up, we will consider interventions to increase employment [129,130], health care access [131], condom use and negotiation skills [132], and mental health and substance use treatment to address epidemic drivers at structural, interpersonal, and individual levels.

### Ethical Considerations

The Johns Hopkins Single Institutional Review Board (IRB00355445) reviewed and approved this study. Johns Hopkins Single Institutional Review Board served as the institutional review board of record for all partner institutions in this multisite study. As a minimal risk study, participants were asked to provide consent in the English or Spanish language using an oral consent form in web-based format prior to initiating research activities. Upon the completion of the survey, participants are provided a US $15 incentive through the modality of their choice. Upon study receipt of participant laboratory results, a US $65 incentive is issued to the participant, for a total of US $80 per visit. Incentives are available through an electronic gift code, which can be redeemed at hundreds of stores on the web, including Walmart, Target, Sephora, Amazon, and other well-known stores, or through a prepaid debit card. The inclusion of a cash-accessible option was critical to the acceptability of the study design by participants, as informed by our prior experiences.

Identifiers were collected from participants for the purposes of shipping specimen collection kits and incentive cards (as applicable) and for study reminders; however, only deidentified data are available for analysis.

## Results

**Figure 4:**
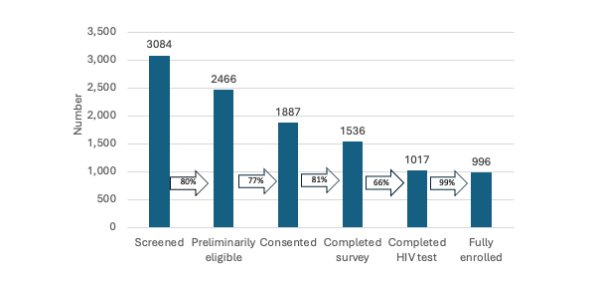
Enrollment continuum in a nationwide, hub-supported cohort of transgender women.

Enrollment opened on March 15, 2023, with enrollment and data collection phases occurring from March 15, 2023, to June 30, 2023, and from November 1, 2023, to December 15, 2023, respectively. As of February 24, 2024, before the next opening of enrollment and data collection, a total of 3084 people were screened for participation. Of those screened, 61.19% (n=1887) were considered eligible and consented to participate in the study, 32.98% (n=1017) completed both the survey and HIV testing activities, and 32.3% (n=996) were fully eligible based on final HIV result and enrolled into the cohort. Figure 4 displays the continuum from screening to enrollment with arrows displaying the percentage remaining from the prior step in the continuum. The most significant drop in the continuum occurred at the completion of the HIV test, in which 66.21% (1017/1536) of those who completed the survey went on to complete a remote HIV test, regardless of the testing method.

Among those enrolled in the cohort, 2.3% (23/996) were enrolled directly in a hub, and 53.6% (534/996) were enrolled through a community-engaged, hub-supported strategy. Recruitment through purely digital recruitment methods, including recruitment through web-based advertisements and dating apps, have contributed to 61.45% (1895/3084) of those screened and 42.7% (425/996) of those enrolled in the cohort.

Table 2 displays the screening characteristics of individuals who completed screening and who ultimately enrolled in the cohort as of February 2024. Between screening and enrollment, ≥5-percentage-point declines were observed in the proportions of participants who were aged 18 to 24 years, who identified as non-Hispanic Black, and who resided in Southern United States.

**Table 2:** Screening characteristics of individuals screened and enrolled into the nationwide, community hub–supported digital cohort.

Characteristic			Screened (N=3084), n (%)		Enrolled (n=996), n (%)
**Age group (y)**					
	18-24	615 (19.94)		146 (14.66)	
	25-29	571 (18.51)		201 (20.18)	
	30-39	1034 (33.53)		367 (36.85)	
	40-49	452 (14.66)		140 (14.06)	
	≥50	381 (12.35)		142 (14.26)	
**Race and ethnic identity**					
	Hispanic Black	72 (2.33)		13 (1.31)	
	Hispanic White	165 (5.35)		65 (6.53)	
	Hispanic and more than 1 or another race	401 (13)		85 (8.53)	
	Non-Hispanic Black	494 (16.02)		111 (11.14)	
	Non-Hispanic White	1562 (50.65)		583 (58.53)	
	Non-Hispanic and more than 1 or another race	230 (7.46)		93 (9.34)	
	Unknown	160 (5.19)		46 (4.62)	
**Language**					
	English	2965 (96.14)		970 (97.39)	
	Spanish	119 (3.86)		26 (2.61)	
**US census region**					
	Midwest	489 (15.86)		187 (18.78)	
	Northeast	748 (24.25)		297 (29.82)	
	Puerto Rico	15 (0.49)		7 (0.7)	
	South	1189 (38.55)		312 (31.33)	
	West	643 (20.85)		193 (19.38)	
**Recruitment source**					
	Community partner	262 (8.5)		63 (6.33)	
	Former participant	536 (17.38)		338 (33.94)	
	Hub	297 (9.63)		133 (13.35)	
	Peer referral	94 (3.05)		37 (3.71)	
	Web-based advertisements	403 (13.07)		77 (7.73)	
	Dating app advertisements	1492 (48.38)		348 (34.94)	

## Discussion

### Anticipated Findings

This study will provide important data on multilevel determinants of HIV risk and syndemic patterns among transgender women at the national level, allowing us to contrast by region and state and better understand the impact of syndemics and contextual factors on HIV prevention and the overall epidemic. The study design builds upon successfully used protocols among transgender women by our study team and aims to address gaps in exclusively digital or exclusively facility-based methods, particularly as gap relates to differences in study population and HIV vulnerabilities [6,39]. This protocol innovatively uses digital cohort methodology supported by community-engaged, hub-supported methods with options for in-person activities to ensure thorough support for and the diversity of experiences among research participants.

Specifically, our preliminary findings have shown that recruitment through purely digital recruitment methods contributed to 61.45% (1895/3084) of those screened to date but only 42.7% (425/996) of those ultimately enrolled in the cohort, highlighting that while digital methods reach geographic breadth, they may not be sufficient alone for achieving HIV recruitment goals or for enrolling a participant population generalizable to the US population of transgender women. As this research proceeds, we will explore how the hybrid community-engaged, hub-supported model expands (or not) the diversity of the sample and supports the retention of the cohort.

### Limitations

The costs and management of this hub-supported model are likely to be perceived as a limitation; however, we suspect that this cohort model is cost-effective, as our experience has shown that individuals disproportionately affected by HIV often need support for study participation and other health and social support. While the pooled contribution from participants enrolling or participating through hubs alongside exclusively web-based participants adds strength to the study, pooled results can provide misleading or overly smoothed inferences. Our analytic “best practices” protocol includes the examination of data to understand the magnitude of heterogeneity in estimates [133]. Further, we will use sensitivity analyses to examine the influence of hubs on specific results, such as by omitting subgroups systematically using jackknife methods with unequal partitions [134]. Finally, we do not propose to trial HIV prevention strategies; however, participants will receive geolocated referrals to HIV prevention and other health or social services tailored to their needs. Further, our use of mathematical modeling provides an opportunity to test multiple HIV prevention strategies among transgender women to inform future trials and national and regional HIV prevention efforts.

### Conclusions

Study findings will have critical implications for the design of future cohorts, given the disparities underlying HIV epidemics. Our understanding of HIV incidence and risk for HIV among transgender women will be augmented by the inclusion of contextual data, which allows us to examine larger social and structural factors that affect the health and well-being of transgender women and can serve as modifiable factors and intervention targets beyond the individual level. The use of mathematical modeling provides an opportunity to simulate multiple HIV prevention strategies among transgender women to inform future trials and national and regional HIV prevention efforts.

## Abbreviations

ART: antiretroviral therapy
CAB: community advisory board
DBS: dried blood spot
EHE: Ending the HIV Epidemic
ENCORE: Enhanced Cohort Methods for HIV Research and Epidemiology
IPV: intimate partner violence
JHU: Johns Hopkins University
PAF: population attributable fraction
PI: principal investigator
PrEP: pre-exposure prophylaxis
PTSD: posttraumatic stress disorder
VOIP: voice over IP

## Data Availability

Deidentified individual data and a data dictionary will be made available upon reasonable request after the approval of a proposal and signing of a data use agreement. There is a formal process for external users to request access to Enhanced Cohort Methods for HIV Research and Epidemiology (ENCORE) data, which involves review and approval by principal investigators from each study site and the community advisory board. Further details and forms can be obtained by emailing ALW and SLR.
